# Significance of Brinkman and Stokes system conjuncture in human knee joint

**DOI:** 10.1038/s41598-022-23402-7

**Published:** 2022-11-08

**Authors:** Nawal Odah Al-Atawi, Shahid Hasnain, Muhammad Saqib, Daoud S. Mashat

**Affiliations:** 1 Department of Mathematics, King Abdulaziz University, Jeddah, Saudi Arabia; 2 Department of Mathematics, University of Chakwal, Chakwal, Pakistan; 3 Department of Mathematics, Khawaja Fareed University of Engineering and Information Technology (KFUEIT), Rahim Yar Khan, Pakistan

**Keywords:** Mathematics and computing, Applied mathematics

## Abstract

Stokes’s equation in the fluid domain and Brinkman’s equation in the porous media are combined in the current study which is designated by the Stokes-Brinkman coupling. The current paper gives a theoretical analysis of the Stokes-Brinkman coupling. It has been shown that such a model is a good match for the knee joint. A flow model has been investigated in order to get a better understanding of the convective diffusion of the viscous flow along the articular surfaces between the joints. The Beavers and Joseph slip conditions which are a specific boundary condition for the synovial fluid are used to solve the governing system of partial differential equations for the synovial fluid and the results are provided here. We develop formulas for the interfacial velocity for both flow through special slip condition and analyse the link between the slip parameters $\alpha$ and $\beta$. Thus, the damping force due to the porous medium naturally when we non-dimensionalize, some parameter which are controlling the structure like, $\beta$ and $\alpha$. Through the development of an analytical solution and numerical simulation (using the finite volume approach) it is hoped that the mechanisms of nutritional transport into the synovial joint will be better understood. According to the data the average concentration has a negative connection with both the axial distance and the duration spent in the experiment. Many graphs have been utilized to gain understanding into the problem’s various characteristics including velocity and concentration, among others. Hyaluronate (HA) is considered to be present in porous cartilage surfaces and the viscosity of synovial fluid fluctuates in response to the amount of HA present.

## Introduction

It is common to refer to joints as the points where two or more bones come together, these points are called “edges” in the context of human skeletons^1^. In order to regulate the range of motion between the linked pieces, each joint has a certain form and structural component. Fibrous joints, cartilaginous joints and synovial joints are the three principal kinds of joints in the human body that enable a wide variety of mobility^1^. These joints are kept in place by connective tissue and are often found in the skulls whereas cartilaginous joints which are somewhat movable, are typically found in the spine or ribs and are hence called, immovable joints^1^. In conclusion the most mobile joints such as synovial joints in human body account for more than half of all joints and are more often found in the limbs and joints of the spine^1^. Having fluid in the synovial joints allows them to move more easily because it helps to minimize friction between the articulating surfaces^1,2^.Some of them are quite static in their movements because they have a lower degree of flexibility than the mobile synovial joints^1,2^. Synovial joints that are not move-able are more stable than synovial joints which are move-able^1,2^. As a result, it is believed that the static synovial joints are more prone to damage than the dynamic synovial joints^1,2^. This is due to the fact that they have different degrees of freedom from one another.

Essentially, each of the synovial joints in the human body has been classified into one of six major groups, which are distinguished by their architecture, composition and forms^2,3^. The pivot joints, saddle joints, hinged joints, ball and socket joints, planar joints and finally condyloid which are most complicated, are among the classes of joints^2,3^. The planar joints are made up of bones that have articulating surfaces which are flat or just slightly curved on one side. Consequently, there is gliding movement between the joints^2,3^. However, the range of motions is restricted since it does not need rotation. Some examples of synovial joints are metacarpal and tarsal bones such as metatarsal joints in the hands and feet^2,3^. There are hinged joints in the ankles, elbows and knee in which one bone moves while the other stays immobile such as door hinged does. It is the only rotational movement that is permitted by pivot joints, which are defined by the fact that their convex articular surfaces are parallel to the longitudinal bone axis^2–4^. Among them are the proximal radio-ulnar joint, the middle atlantoaxial joint and the distal radio-ulnar joint. The most common kind of joint known as condyloid joint permits angular movement along two axes^2–4^. Consider the joint in the wrist and the finger which can move both side to side and up and down, as two examples of joints that move in this manner. The oval-shaped end of one bone fits into a similarly oval-shaped hollow on the other bone which allows the bones to move together. Some writers refer to it as ellipsoidal joints in their books^3,4^.

The saddle joint is a form of joint that allows angular motion comparable to that of the condyloid joint, however, the saddle joint has a broader range of motion than the condyloid joint. Saddle joints are formed anatomically by two bones that are concave on one side and convex on the other, as in the thumb joint, which has more freedom of movement than the wrist or finger, and has the ability to move back and forth, as well as up and down, in comparison to other joint^3,4^. There are greatest degrees of flexibility of any kind of joint in all synovial joints which includes ball and socket joints, allowing movement in all directions. A rounded ball-like structure is present on one end of the bone which fits into a cup-like socket on the other end of the bone in this kind of joint. For example, the shoulder and hip joints, are examples of such joints^3,4^. The shoulder, hip and knee have the most degrees of flexibility out of all the synovial joints, making them more prone to the dangers of accidents as well as varying degrees of arthritis which typically affects the synovial joint and produces impairment^3,4^. In the medical world, arthritis is defined as inflammation of the joints. Osteoarthritis is a condition that is known to decrease joint function, causing pain and as it progresses from its early stages to its advanced stages, it may eventually result in disability^3,4^.

**Figure 1:**
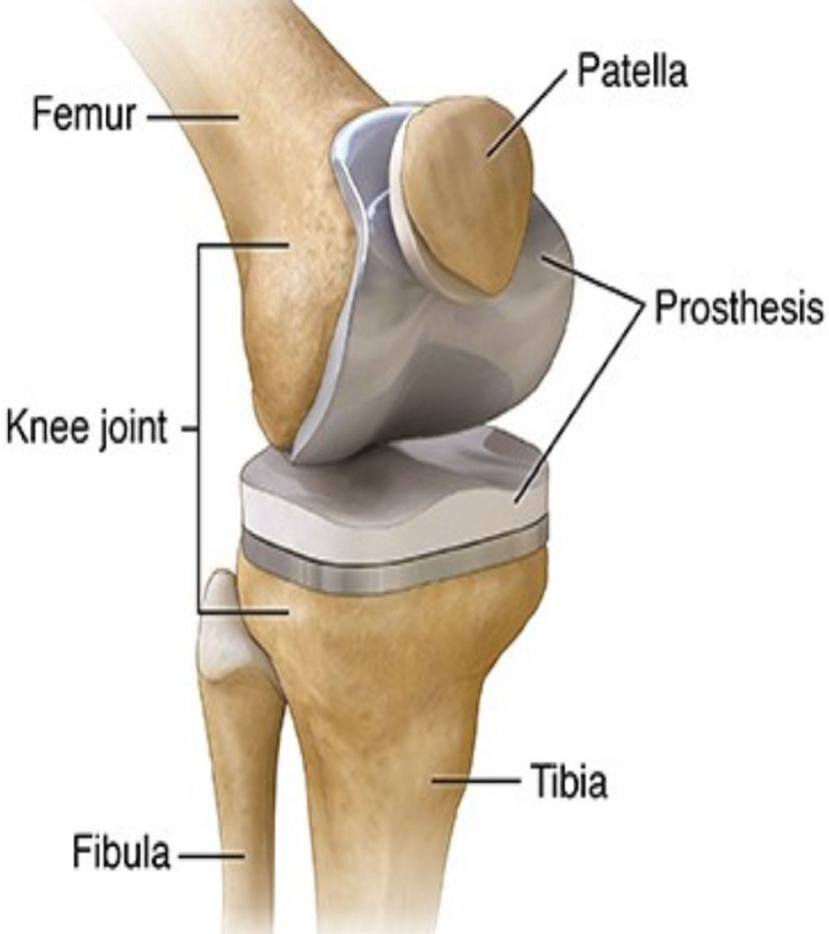
Shows physical information of Synovial joint^5^ (Human Knee joint).

When it comes to joints, the knee joint is the most complex in addition to being essential in the human body. In the skeletal system, it is made up of two long bones. The femur( also known as thigh bone) and the tibia (sometimes known as the shin bone) are the two bones that make up the lower leg, see Fig. 1^3–5^. The knee joint is responsible for a variety of functions. It is possible for the knee to be loaded up to seven times the body weight while doing intense activities like walking, running and jumping^3–5^. The knee joint is made up of two articulations which are, a medial and lateral tibia femoral articulation, as well as a patella femoral articulation, which are located on each side of the tibia.

Hyaline cartilage covers the articulating surface of each bone and it is the most essential component of the knee joint because it provides the joint with an extraordinarily smooth surface and protects the underlying bones from injury^3,4^. An internal shock absorber inside the knee joint, the meniscus, is located in the space between the two bones that make up the joint. When performing hard exercises, the meniscus helps to prevent the knee bones from colliding with one another^3,4^. A key contribution of modelling of the knee joint is that, it gives information which is difficult to get via testing and it also allows for the production of realistic models that may be utilized to better surgical and rehabilitation treatments. Equilibrium of forces and moments coming from external loads may be solved by evaluating the influence of individual components and predicting their behaviour rather than calculating the equations. Different types of models may be used to present data, including dynamical system, statistical models, differential equations (including both ordinary differential equations (ODEs) and partial differential equations (PDEs)) and theoretical models.

In light of previous research, the purpose of this article is to investigate the Stokes equation in the fluid domain and the Brinkman equation in the porous media, both of which are designated by the Stokes Brinkman coupling under the Beavers and Joseph slip conditions, which are a specific boundary condition for the synovial fluid. After the governing equations have been modelled, the appropriate similarity transformations are used in order to change the equations into forms that are self similar. Take note of the fact that such solutions are very uncommon when there is viscous dissipation present. In order to validate the reliability of our findings, we used the Finite Volume technique to find numerical solutions to the resulting self similar equations. In addition, the effects that several relevant factors have on the dimensionless velocity and temperature are depicted and explored in this article.

## Human knee joint modelling using mathematical equations

The most frequently used joint which is the knee joint in the human body and even a very little problem with the knee joint will most certainly have an influence on a person’s ability to do their daily tasks normally. Because of the vital role that the knee joints play in human mobility as well as the fact that they are a major load bearing joint, the important of the knee can not be stressed at any point of the time^3,4,6^. The knee joint is the biggest weight bearing joint in the human skeletal system, according to the American Academy of Orthopedic Surgeons, in addition, it is the most complex joint in the human skeletal system^4^. This difficulty may manifest itself in a variety of ways, including composition, function, geometry, and set-up. To this point, we have concentrated only on the description of the joints, selecting the most appropriate model for synovial fluid that can accurately reproduce the experimental data throughout a given range of deformations^3,4,6^. For the time being, let us develop the balancing laws that describe the flow of synovial fluid with some justification for simplifications. Let us bring these simplifications to a close before we begin putting down the explicit equations^3,4,6^.

### Balance law

In case of synovial fluid flow, it is feasible to characterise it in term of the pressure field ${\mathbb {P}}$ and the velocity field ${\mathbb {V}}$, both of which are regulated by the Naiver Stokes equations with the constraint of incomprehensibility as well as the pressure^6–8^. Porous fluids are defined as fluids in porous media in which the movement of momentum within the fluid as a consequence of shear stresses plays an essential role and in which the movement of momentum within the fluid is crucial^7,8^. Darcy’s law introduces a term into the momentum balance that takes into consideration viscous transport in this mathematical model which considers the pressure as an independent variable in addition to the pressure and flow velocity vector^9,10^. Convection diffusion defines the concentration distribution of a flow when it is applied to it^9,10^. The concentration distribution is represented by the scalar field ${\mathbb {C}}$^9–12^. The system of governing equations has the following representation:

- *Viscous Newtonian fluids theory* Simply extending the basic theory of viscous fluid flow, it accurately predicts the flow behavior of fluids that have a substructure, such as synthetic lubricants, liquid crystals, synovial fluid, and even animal blood^12–14^. The mechanical interactions in a fluid medium are described in such a manner that the idea of couple stresses is introduced, rather than symmetric. Modelling synovial fluid using Stokes theory (1966) is utilized in order to establish connections with the momentum and continuity equations^12–14^. Equations that are defined in this theory include the following:
  1 $$\begin{aligned}{} & {} \rho \dfrac{D{\mathbb {V}}}{D t}=- \triangledown {\mathbb {P}}+\mu \triangledown {\mathbb {C}}, \end{aligned}$$
  2 $$\begin{aligned}{} & {} \quad {\mathbb {Q}} = \rho {\mathbb {F}} + \dfrac{1}{2} \rho \triangledown \times {\mathbb {C}} - \eta \triangledown ^{4} {\mathbb {V}}, \end{aligned}$$
  3 $$\begin{aligned}{} & {} \quad \triangledown \cdot {\mathbb {V}} = 0. \end{aligned}$$
  where $\rho$ is density of synovial fluid, *D*/*Dt* is a total derivative, vectors ${\mathbb {V}}$, ${\mathbb {F}}$ and ${\mathbb {C}}$ represent velocity, body force and body couple per unit mass. $\mu$ is viscosity and $\eta$ represents coupled stress constant^12–14^.
- *Porous media Brinkman viscosity mode* Inter-facial flow between a free fluid and a porous medium is well represented by the Brinkman’s equation. A Brinkman viscosity can still be determined but the pore scale flow is still not known^13–16^. An a priory estimate of effective viscosity for rough porous media can be determined from the interface slip length and the interior permeability for slip and permeability from unit cell analysis. For irregular porous structures, the Brinkman’s model physical origins show that it is much more accurate^14–17^.
  4 $$\begin{aligned} - \triangledown {\mathbb {P}} + \mu ' \triangledown ^{2} {\mathbb {V}}= \dfrac{\mu }{{\mathbb {K}}}{\mathbb {V}}. \end{aligned}$$
  where ${\mathbb {V}}$ is seepage velocity of the fluid, $\mu$ is dynamic viscosity, $\mu '$ is effective viscosity of the fluid inside the porous medium and ${\mathbb {K}}$ permeability of the porous medium^14–16^.
- *Power law model* The relation related to shear rate and viscosity in a steady shear flow called as Power law model (Ostwald de Waele relationship) can be written as^16,18^:
  5 $$\begin{aligned} \mu= & {} K ({\dot{\gamma }})^{n-1} \end{aligned}$$
  6 $$\begin{aligned} \mu= & {} K \left( \dfrac{\partial u}{\partial y} \right) ^{n-1}, \end{aligned}$$
  where $\mu$ is viscosity, ${\dot{\gamma }}$ shear rate, *K* and *n* are curve fitting coefficients^16–18^.

### Kinematics

The conservation of laws of physics are used in fluid flow mathematical equations;

- In the fluid, fluid mass is preserved (continuity equation)^18^.
  $$\begin{aligned} \dfrac{\partial \rho }{\partial t} + {\text{div}} \ (\rho {\mathbb {V}}) = 0, \end{aligned}$$

in which the time dependent density variation is represented by the first term while the net mass out of the fluid element by its boundaries, is shown by 2nd term which is refereed to as the convective term^18^. Our assumptions are, the density is constant (in-compressible flow), steady and two dimensional which can be written as,

7 $$\begin{aligned} \dfrac{\partial u}{\partial x}+\dfrac{\partial v}{\partial y} = 0. \end{aligned}$$

- The rate of change of momentum equals the sum of the forces on a fluid particle (Newton’s 2nd law of motion)^18^.

Conservation of momentum equation in components form, in x- direction is defined as,

$$\begin{aligned} \dfrac{\partial u}{\partial t} + u \dfrac{\partial u}{\partial x} + v \dfrac{\partial u}{\partial y} = \dfrac{\partial }{\partial x}(-{\mathbb {P}} + \tau _{xx})+\dfrac{\partial }{\partial y}( \tau _{yx})+S_{M_{x}}, \end{aligned}$$

Conservation of momentum equation in components form, in y- direction is defined as,

$$\begin{aligned} \dfrac{\partial v}{\partial t} + u\dfrac{\partial v}{\partial x} + v\dfrac{\partial v}{\partial y} = \dfrac{\partial }{\partial x}(\tau _{xy})+\dfrac{\partial }{\partial y}(-{\mathbb {P}} + \tau _{yy})+S_{M_{y}}, \end{aligned}$$

where $\tau _{xx}$ and $\tau _{yy}$ are viscous stress components, linear deformation rate as well as volumetric deformation rate are included^17,18^. Because liquids are in-compressible so the mass conservation equation is continuous and viscous stresses are twice the local rate of linear deformation times the dynamic viscosity^18–21^.

$$\begin{aligned} \tau _{xx}= & {} 2\mu \dfrac{\partial u}{\partial x} + \lambda \ \ div \ {\mathbb {V}}, \\ \tau _{yy}= & {} 2\mu \dfrac{\partial v}{\partial y} + \lambda \ \ div \ {\mathbb {V}}, \\ \tau _{xy}= & {} \tau _{yx}= \mu \left( \dfrac{\partial u}{\partial y} + \dfrac{\partial v}{\partial x} \right) . \end{aligned}$$

Also, $S_{M_{x}}$ and $S_{M_{y}}$ are source terms include contribution due to body forces only and ${\mathbb {P}}$ is pressure^18–21^. We consider $S_{M_{x}}=S_{M_{y}}=0$ with linear deformation rate and neglect the variation of pressure normal to very thin film of lubrication^18–21^. We know that, *n* is power law index with $n=1$ for Newtonian fluids. Above assumptions led to the following expression,

8 $$\begin{aligned} 0= & {} - \dfrac{\partial {\mathbb {P}}}{\partial x} + \mu \dfrac{\partial }{\partial y}[\Vert \dfrac{\partial u}{\partial y}\Vert ^{n-1}\dfrac{\partial u}{\partial y}], \end{aligned}$$

9 $$\begin{aligned} 0= & {} - \dfrac{\partial {\mathbb {P}}}{\partial y}. \end{aligned}$$

- It is a combination of linear momentum and mass conservation for the fluid in large pores and flow channels as well as Darcy’s equation for regions with unresolved pores that gives rise to the Brinkman’s equation, refer to Eq. (4).

The consideration of unidirectional motion with $\mu ' = \mu$ in conjunction with the continuity equation results in the following equation,

10 $$\begin{aligned} \dfrac{\mu }{{\mathbb {K}}} \ u= & {} -\dfrac{\partial {\mathbb {P}}}{\partial x}+ \mu ' \dfrac{\partial ^{2} u}{\partial \ y^{2}}. \end{aligned}$$

Let us consider a special slip boundary condition at upper permeable surfaces,

11 $$\begin{aligned} u = - \dfrac{\sqrt{{\mathbb {K}}}}{\alpha } \ \dfrac{\partial u}{\partial y}, \ \ \ at \ \ \ y = h. \end{aligned}$$

where ${\mathbb {K}}$ and $\alpha$ are permeability of porous medium and slip parameter respectively^18–21^. The boundary of porous medium with a free viscous fluid is the continuity of pressure and the normal mass flux which give rise to wipe up the tangential velocity of the synovial fluid^18–21^. Such boundary condition was proposed by Beavers and Joseph (BJ) in 1967 as a empirical formula^19,20,22^. The analysis showed that BJ condition is macroscopic law which describe a phenomena at a macroscopic scale whereas characteristic length is much larger than the pore characteristic size^19,20,22^. Symmetry boundary condition will be defined as,

12 $$\begin{aligned} \dfrac{\partial u}{\partial y}=0, \ \ \ at \ \ \ y = 0. \end{aligned}$$

The hyaluronic acid (HA) molecules in synovial fluid linked by convection diffusion phenomena is simple mass balance between changes in concentration ${\mathbb {C}}$ of solutes^21–24^,

13 $$\begin{aligned} \dfrac{\partial {\mathbb {C}}}{\partial t}+(u-{\bar{u}})\dfrac{\partial {\mathbb {C}}}{\partial x}+(v-{\bar{v}})\dfrac{\partial {\mathbb {C}}}{\partial y}={\mathbb {D}}_{Diff} \left( \dfrac{\partial ^{2} {\mathbb {C}}}{\partial x^{2}}+\dfrac{\partial ^{2} {\mathbb {C}}}{\partial y^{2}} \right) -{\mathbb {R}}{\mathbb {C}}. \end{aligned}$$

where ${\mathbb {C}}$, ${\mathbb {R}}$ and ${\mathbb {D}}_{Diff}$ are concentration of solutes, chemical reaction with first order rate (such reaction occurs in fully developed flow after diffusion) and diffusion constant respectively^21–24^.

### Synovial fluid governing system

**Figure 2:**
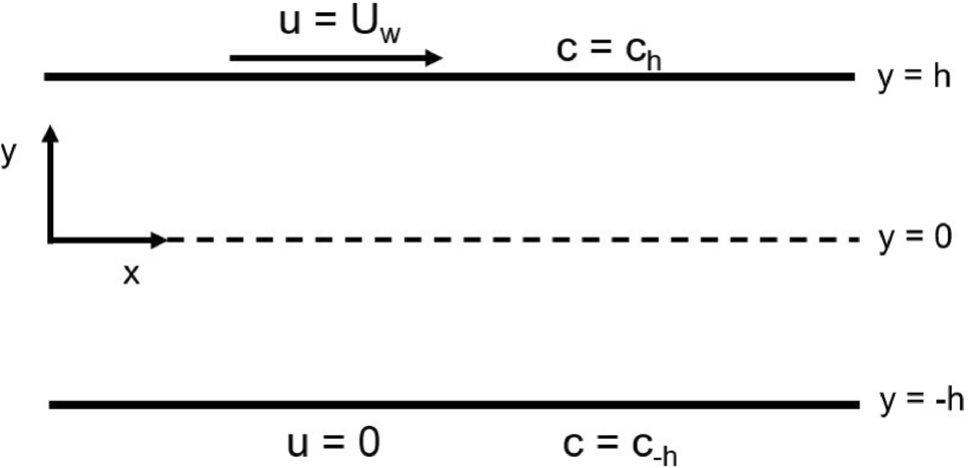
Shows physical configurations to knee joint in which synovial fluid is confined between two infinitely long parallel plates apart by a distance of 2*h* with the top plate is moving with the help of BJ condition.

According to aforementioned description, the proposed research problem for analysis is physical configured of two rectangular palates of infinite length in *x* direction, which can be seen from Fig. 2. The synovial fluid (film) of thickness 2*h* separates the two surfaces from one another. Also, *u* and *v* are the components of the synovial fluid velocity along *x* and *y* directions respectively. The synovial fluid has been represented as viscoelastic fluid and its elasticity is important in joint lubrication. It is surrounded by porous layer which represent synovial membrane. We introduce the conventional assumption of lubrication theory into the Navier-Stokes (NS) equation of motion and ignore the fluctuation of pressure corresponding to a very thin layer of lubrication in the NS equations. The pressure ${\mathbb {P}}$ in the fluid film region along the NS equations which is set up with Beavers and Joseph boundary condition on upper plate and symmetry condition at $y=0$, we have the following equations,

14 $$\begin{aligned} 0= & {} - \dfrac{\partial {\mathbb {P}}}{\partial x} + \mu \dfrac{\partial ^{2} u}{\partial y^{2}}, \ \ for \ \ u = - \dfrac{\sqrt{{\mathbb {K}}}}{\alpha } \ \dfrac{\partial u}{\partial y}, \ \ at \ \ y = h, \end{aligned}$$

15 $$\begin{aligned}{} & {} \dfrac{\partial u}{\partial y} = 0, \ \ at \ \ y = 0, \end{aligned}$$

16 $$\begin{aligned}{} & {} - \dfrac{\partial {\mathbb {P}}}{\partial y} = 0. \end{aligned}$$

The Brinkman Eq. (10) set up with Beavers and Joseph boundary condition on upper plate and symmetry condition at $y=0$, we have the following equations,

17 $$\begin{aligned} \dfrac{\mu }{{\mathbb {K}}} \ u= & {} -\dfrac{\partial {\mathbb {P}}}{\partial x}+ \mu ' \dfrac{\partial ^{2} u}{\partial \ y^{2}}, \ \ at \ \ u = - \dfrac{\sqrt{{\mathbb {K}}}}{\alpha } \ \dfrac{\partial u}{\partial y}, \ \ at \ \ y = h, \end{aligned}$$

18 $$\begin{aligned} \dfrac{\partial u}{\partial y} = 0, \ \ at \ \ y = 0. \end{aligned}$$

The concentration Eq. (13) is build up with the following assumptions:

- Diffusion process associated with steady convection.
- The rate of longitudinal diffusion is much lower than the rate of transverse diffusion.
  19 $$\begin{aligned} \implies \dfrac{\partial ^{2} {\mathbb {C}}}{\partial y^{2}}>> \dfrac{\partial ^{2} {\mathbb {C}}}{\partial x^{2}}, \end{aligned}$$

20 $$\begin{aligned} (u-{\bar{u}})\dfrac{\partial {\mathbb {C}}}{\partial x} = {\mathbb {D}}_{Diff} \left( \dfrac{\partial ^{2} {\mathbb {C}}}{\partial y^{2}} \right) - {\mathbb {R}} {\mathbb {C}}, \end{aligned}$$

where, ${\bar{u}}$ is the cross-sectional average velocity.

21 $$ \begin{aligned} {\mathbb {C}}(x,y) = c_{0}, \ \ at \ \ y = h \ \  \&  \ \ {\mathbb {C}}(x,y) = c_{1}, \ \ at \ \ y = -h. \end{aligned}$$

## Methodology

Partial differential equations may be found in practically every discipline of research and engineering, including mathematics. Only a small number of these instances can be solved analytically to a high degree of precision. It is almost always necessary to use a numerical technique in order to answer the challenges. Non-dimensionalization may also be used to recover the system’s core properties, which might be very useful in certain cases. The method is especially advantageous for system that may be representated by partial differential equations. For numerical solution to aforementioned system, we used finite volume method.

### Nondimensionalization

#### Velocity distribution profile for Eq. (14)

We define the non-dimensional quantities,

22 $$\begin{aligned} x^{*}=\dfrac{x}{H}, \ y^{*}=\dfrac{y}{H}, \ u^{*}=\dfrac{u}{{\bar{u}}}, \ \mu _{rel} = \dfrac{\mu }{\mu '}, p^{*}=\dfrac{{\mathbb {P}}}{\mu {\bar{u}}/H}, \ {\mathbb {D}}a=\dfrac{{\mathbb {K}}}{H^{2}}, \ \beta ^{2}=\dfrac{1}{{\mathbb {D}}a} \end{aligned}$$

Apply non-dimensionalization technique to Eqs. (14, 15, 16), we have the following,

23 $$\begin{aligned}{} & {} \mu \ \dfrac{{\bar{u}} \ \partial ^{2} \left( \dfrac{u}{{\bar{u}}} \right) }{H^{2}\partial \left( \dfrac{y}{H} \right) ^{2}}= \left( \dfrac{\mu \ {\bar{u}}}{H} \right) \dfrac{\dfrac{\partial {\mathbb {P}}}{\mu {\bar{u}}/H}}{H \ \partial \left( \dfrac{x}{H} \right) }, \end{aligned}$$

24 $$\begin{aligned}{} & {} \quad \dfrac{\partial ^{2} u^{*}}{\partial {y^{*}}^{2}}=\dfrac{\partial p^{*}}{\partial x^{*}}, \end{aligned}$$

Non-dimensionalised boundary conditions:

25 $$\begin{aligned} u^{*}=-\dfrac{\sqrt{{\mathbb {D}}a}}{\alpha } \ \dfrac{\partial u^{*}}{\partial y^{*}}, \ \ at \ \ y^{*}=1, \ \dfrac{\partial u^{*}}{\partial y^{*}}=0, \ \ at \ \ y^{*}=0. \end{aligned}$$

Using simplified notation, the solution for the velocity profile can be expressed as follows:

26 $$\begin{aligned} u^{*}=\dfrac{\partial p^{*}}{\partial x^{*}} \left( \dfrac{{y^{*}}^{2}}{2}-\dfrac{1}{2}-\dfrac{\sqrt{{\mathbb {D}}a}}{\alpha } \right) \end{aligned}$$

#### Velocity distribution profile for Eq. (17)

By applying non-dimensional quantities (22) to Eqs. (17, 18), we have the following expression,

27 $$\begin{aligned}{} & {} \mu ' \ \dfrac{{\bar{u}} \ \partial ^{2} \left( \dfrac{u}{{\bar{u}}} \right) }{H^{2}\ \partial \left( \dfrac{y}{H} \right) ^{2}}-\dfrac{\mu }{{\mathbb {K}}} \ {\bar{u}} \ \dfrac{u}{{\bar{u}}}= \left( \dfrac{\mu \ {\bar{u}}}{H}\right) \dfrac{\dfrac{\partial {\mathbb {P}}}{\mu {\bar{u}}/H}}{H \ \partial \left( \dfrac{x}{H}\right) }, \end{aligned}$$

28 $$\begin{aligned}{} & {} \quad \dfrac{\partial ^{2} u^{*}}{\partial {y^{*}}^{2}}-\dfrac{\mu }{\mu '} \ \dfrac{H^{2}}{{\mathbb {K}}} \ u^{*}=\dfrac{\mu }{\mu '} \ \dfrac{\partial p^{*}}{\partial x^{*}}. \end{aligned}$$

29 $$\begin{aligned}{} & {} \quad \dfrac{\partial ^{2} u^{*}}{\partial {y^{*}}^{2}}-\mu _{rel} \ \beta ^{2} \ u^{*}=\mu _{rel} \ \dfrac{\partial p^{*}}{\partial x^{*}}, \end{aligned}$$

By utilising Eq. (25) and with some simplified notation, the solution for the velocity profile can be expressed as follows:

30 $$\begin{aligned} u^{*}=\dfrac{1}{\mu _{rel} \ \beta ^{2}} \dfrac{\partial p^{*}}{\partial x^{*}} \left[ \dfrac{cosh \left( \mu _{rel} \ \beta ^{2} \right) \ y^{*}}{cosh \left( \mu _{rel} \ \beta ^{2} \right) + \mu _{rel} \ \beta ^{2} \ \dfrac{\sqrt{{\mathbb {D}}a}}{\alpha } \ sinh \left( \mu _{rel} \ \beta ^{2} \right) } -1 \right] \end{aligned}$$

For sake of simplicity, we can omit the star sign from Eqs. (26), (30) to find the average value of the velocity, in the following way,

31 $$\begin{aligned} {\bar{u}} = \dfrac{1}{2h} \int _{-h}^{h} u \,dy \end{aligned}$$

#### Concentration distribution profile

Apply non-dimensionalization technique to Eqs. (20, 21), such as

32 $$ \begin{aligned} {\mathbb {C}}^{\star }= \dfrac{{\mathbb {C}}}{c_{0}}, \ \ y^{*}= \dfrac{y}{H}, \ \ X= \dfrac{x}{{\bar{u}} L}, \ \ {t}^{\star }= \dfrac{t}{{\bar{t}}},\ \xi = \dfrac{X-{\bar{u}} t}{L}, \ \  \&  \ \ {\gamma }^{2}=\dfrac{H^{2}{\mathbb {R}}}{D_{Diff}}. \end{aligned}$$

which led to the following equation,

33 $$\begin{aligned} \left. \begin{aligned} \dfrac{\partial ^{2}{\mathbb {C}}^{\star }}{\partial {y*^{2}}}-{\gamma }^{2}{\mathbb {C}}^{\star }={\mathbb {T}}u^{\star }, \ \ {\mathbb {T}}=\dfrac{H^{2}}{ L D_{Diff}}\dfrac{\partial {\mathbb {C}}^{\star }}{\partial \xi } \end{aligned}\right\} \end{aligned}$$

Solution to problem (33) is,

34 $$\begin{aligned} {\mathbb {C}}^{\star } = \dfrac{1}{Cosh \gamma } \left[ \dfrac{(c_{0}+c_{1})}{2}+\dfrac{{\mathbb {T}}u^{\star }}{{{\gamma }^{2}}} \right] Cosh \gamma y^{*} + \dfrac{1}{Sinh \gamma } \left[ \dfrac{(c_{0}-c_{1})}{2} \right] Sinh \gamma y^{*} - \dfrac{1}{{\gamma }^{2}}{\mathbb {T}}u^{\star }. \end{aligned}$$

### Finite volume method

There are many computational techniques that are most often employed in computational fluid dynamics. These include finite difference, finite volume, finite element and their variations. The current study focuses mostly on the finite volume approach for computing solutions to the aforementioned systems (14, 15, 16 and 17). The following are the main steps involved in putting method into action:

- Divide the domain into the sub-domains of finite size(finite control volumes) by a finite number of grid points (like Nodes)^18,22–24^,
- Over each sub-domain, integrate the governing differential equation (GDE)^18,22–24^,
- An interpolation function can be considered as a profile assumption for the dependent variable to evaluate the integral expression which give an algebraic quantity at the grid points^18,22–24^.

#### Velocity profile algorithm using Eqs. (14, 17)

Formal integration of the governing equation over a control volume gives the following expression,

35 $$\begin{aligned}{} & {} \int _{\bigtriangleup V} u_{yy} \ dV + \int _{\bigtriangleup V} Source \ dV=0, \end{aligned}$$

36 $$\begin{aligned}{} & {} \quad \left[ A \dfrac{du}{dy} \right] _{n}- \left[ A \dfrac{du}{dy} \right] _{s} + Source \ \triangle V= 0. \end{aligned}$$

37 $$\begin{aligned} A_{n} \dfrac{(u_{N}-u_{P})}{\delta _{y_{PN}}}- A_{s} \dfrac{(u_{P}-u_{S})}{\delta _{y_{SP}}} + Source \ A \delta y= 0. \end{aligned}$$

When source term is represented in the linearised form,

$$\begin{aligned} Source \triangle V = S_{u}+S_{P}u_{P} \end{aligned}$$

Equation (37) can be rearranged as;

38 $$\begin{aligned} \left( \dfrac{A_{n}}{\delta _{y_{PN}}}+\dfrac{A_{s}}{\delta _{y_{SP}}} \right) u_{P}= \dfrac{A_{n}}{\delta _{y_{PN}}}u_{N} + \dfrac{A_{s}}{\delta _{y_{SP}}}u_{S}+ S_{u}+S_{P}u_{P} \end{aligned}$$

Nodes at interior:

39 $$\begin{aligned} a_{P}u_{P} = a_{S}u_{S}+a_{N}u_{N}+ S_{u}, \ a_{S}=\dfrac{A_{s}}{\delta \eta _{SP}},\ a_{N}=\dfrac{A_{n}}{\delta \eta _{PN}},\ a_{P}=a_{S}+a_{N}-S_{P}, \end{aligned}$$

where $\delta \eta _{SP}=\delta \eta _{PN}=\delta y$, which represents the distance between any two nodes. Also, $A_{s}=A_{n}=A$.

Nodes at le f t boundary:

 For left boundary node, we use $u=0$ at $y =0$.

40 $$\begin{aligned}{} & {} \dfrac{A}{\delta y}(u_{N}-u_{P})- \dfrac{2A}{\delta y}(u_{P}-u_{a})+Source_{y} \ A \ \delta y=0 \end{aligned}$$

41 $$\begin{aligned}{} & {} a_{P}u_{P} = a_{S}u_{S}+a_{N}u_{N}+ S_{u}, \ a_{S}=0,\ a_{N}=\dfrac{A}{\delta y},\ a_{P}=a_{S}+a_{N}-S_{P},\ S_{P}=-\dfrac{2A}{\delta y},\ S_{u}=\dfrac{2A}{\delta y} \ u_{a}+Source_{y} \ A \ \delta y. \end{aligned}$$

Nodes at right boundary:

For right boundary node, we have the following expression;

42 $$\begin{aligned}{} & {} A \ \left( - \dfrac{\alpha h}{\sqrt{{\mathbb {K}}}} u_{P} \right) - \dfrac{A}{\delta y} \left( u_{P}-u_{S} \right) +Source_{y} \ A \ \delta y=0 \end{aligned}$$

43 $$\begin{aligned}{} & {} a_{P}u_{P} = a_{S}u_{S}+a_{N}u_{N}+ S_{u}, \ a_{S}=\dfrac{A}{\delta y},\ a_{N}=0,\ a_{P}=a_{S}+a_{N}-S_{P},\ S_{P}=A \ (- \dfrac{\alpha h}{\sqrt{{\mathbb {K}}}}),\ S_{u}=Source_{y} \ A \ \delta y. \end{aligned}$$

#### Concentration profile algorithm using Eq. (20)

Now we apply FVM to Concentration Eqs. (20 which can be defined as;

44 $$\begin{aligned}{} & {} \int _{\bigtriangleup V} {\mathbb {C}}_{yy} \ dV + \int _{\bigtriangleup V} \gamma ^{2} \ {\mathbb {C}} \ dV + \int _{\bigtriangleup V} Source_{{\mathbb {C}}} \ dV=0, \end{aligned}$$

45 $$\begin{aligned}{} & {} \left[ A \dfrac{d{\mathbb {C}}}{dy} \right] _{n}- \left[ A \dfrac{d{\mathbb {C}}}{dy} \right] _{s} + Source \ \Delta V= 0. \end{aligned}$$

46 $$\begin{aligned}{} & {} \quad A_{n} \dfrac{\left( {\mathbb {C}}_{N}-{\mathbb {C}}_{P} \right) }{\delta _{y_{PN}}}- A_{s} \dfrac{({\mathbb {C}}_{P}-{\mathbb {C}}_{S})}{\delta _{y_{SP}}} + Source \ A \delta y= 0. \end{aligned}$$

Equation (46) can be rearranged as;

47 $$\begin{aligned} \left( \dfrac{A_{n}}{\delta _{y_{PN}}}+\dfrac{A_{s}}{\delta _{y_{SP}}} \right) {\mathbb {C}}_{P}= \dfrac{A_{n}}{\delta _{y_{PN}}}{\mathbb {C}}_{N} + \dfrac{A_{s}}{\delta _{y_{SP}}}{\mathbb {C}}_{S}+ S_{u}+S_{P}{\mathbb {C}}_{P} \end{aligned}$$

Nodes at interior

48 $$\begin{aligned} a_{P}{\mathbb {C}}_{P} = a_{S}{\mathbb {C}}_{S}+a_{N}{\mathbb {C}}_{N}+ S_{{\mathbb {C}}}, \ a_{S}=\dfrac{A}{\delta y},\ a_{N}=\dfrac{A}{\delta y},\ a_{P}=a_{S}+a_{N}-S_{P},\ S_{P}=-\gamma ^{2} \ A \ \delta y,\ S_{{\mathbb {C}}}= A \ Source_{{\mathbb {C}}} \ \delta y \end{aligned}$$

Nodes at left boundary:

For left boundary node, we use ${\mathbb {C}}=c_{0}$ at $y =1$.

49 $$\begin{aligned} \dfrac{A}{\delta y}({\mathbb {C}}_{N}-{\mathbb {C}}_{P})- \dfrac{2A}{\delta y}({\mathbb {C}}_{P}-{\mathbb {C}}_{a})+Source_{{\mathbb {C}}} \ A \ \delta y -\gamma ^{2} \ A \ \delta y \ {\mathbb {C}}_{P}=0 \end{aligned}$$

which suggests;

50 $$\begin{aligned} a_{P}{\mathbb {C}}_{P} = a_{S}{\mathbb {C}}_{S}+a_{N}{\mathbb {C}}_{N}+ S_{{\mathbb {C}}},a_{S}=0,a_{N}=\dfrac{A}{\delta y},a_{P}=a_{S}+a_{N}-S_{P},S_{P}=-\dfrac{2A}{\delta y}-\gamma ^{2} \ A \ \delta y,S_{u}=\dfrac{2A}{\delta y} \ {\mathbb {C}}_{a}+Source_{{\mathbb {C}}} \ A \ \delta y. \end{aligned}$$

Nodes at right boundary:

For right boundary node, we have the following expression ${\mathbb {C}}=c_{1}$ at $y =-1$.

51 $$\begin{aligned} \dfrac{2A}{\delta y}({\mathbb {C}}_{b}-{\mathbb {C}}_{P})- \dfrac{A}{\delta y}({\mathbb {C}}_{P}-{\mathbb {C}}_{S})+Source_{{\mathbb {C}}} \ A \ \delta y -\gamma ^{2} \ A \ \delta y \ {\mathbb {C}}_{P}=0 \end{aligned}$$

which suggests;

52 $$\begin{aligned}{} & {} a_{P}{\mathbb {C}}_{P} = a_{S}{\mathbb {C}}_{S}+a_{N}{\mathbb {C}}_{N}+ S_{{\mathbb {C}}},a_{S}=\dfrac{A}{\delta y},a_{N}=0,a_{P}=a_{S}+a_{N}-S_{P}, \nonumber \\{} & {} S_{P}=-\dfrac{2A}{\delta y}-\gamma ^{2} \ A \ \delta y,S_{u}=\dfrac{2A}{\delta y} \ {\mathbb {C}}_{b}+Source_{{\mathbb {C}}} \ A \ \delta y. \end{aligned}$$

## Results and discussion

**Figure 3:**
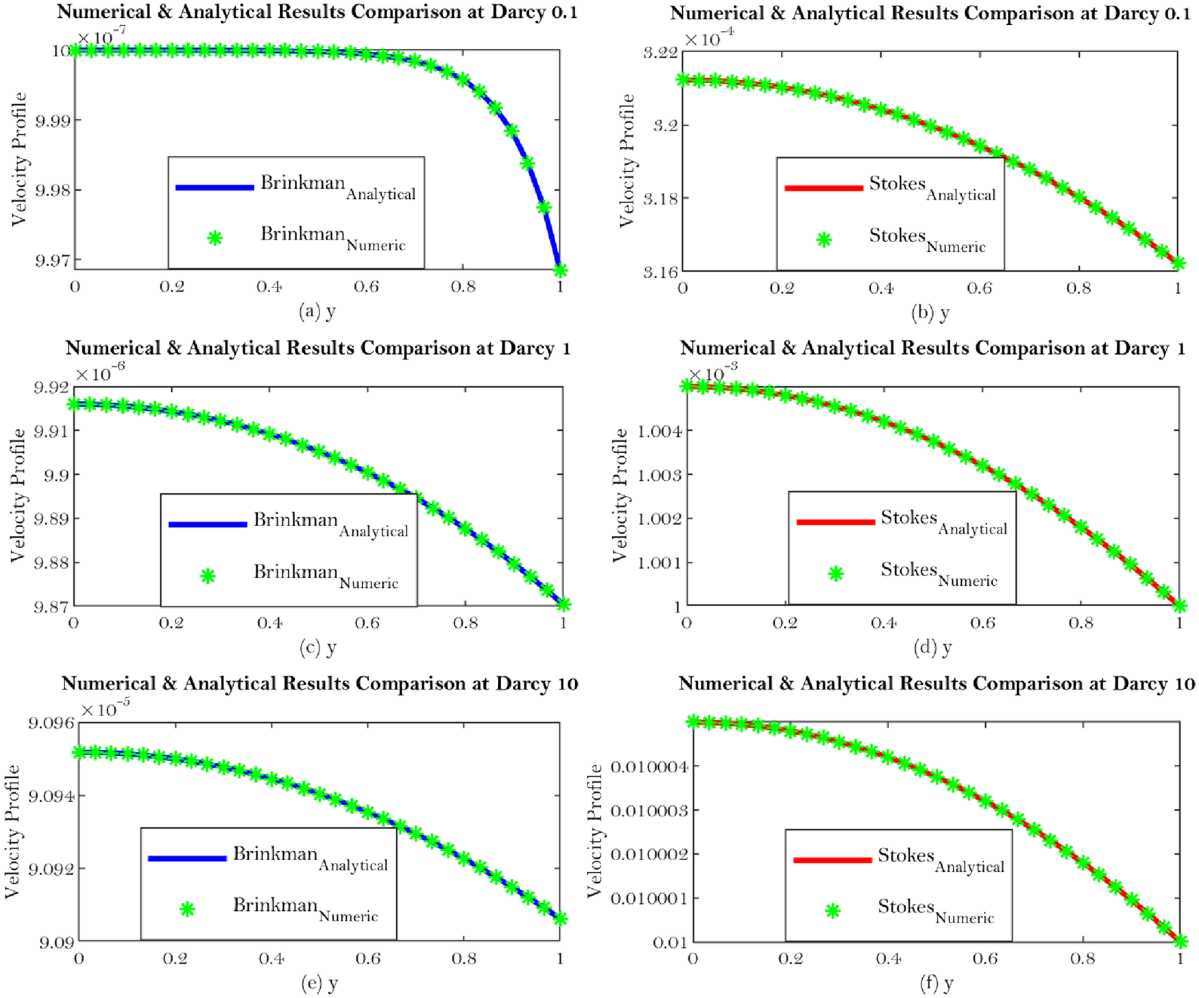
Shows the general solution comparison corresponding to the interface boundary condition which can be seen from Eqs. (14, 15).

One length scale involved which is spacing between two plates so we introduce non-dimensionalization which is lead by *x* and *y* both are normalized by *H*. Then $u^{*}$ is a characteristic velocity, ${\bar{u}}$ is the mean velocity and the $p^{*}$ is the pressure so the corresponding non dimensionalization is discussed for Synovial fluid flow in Knee joints. Such nondimenionalization lead by an additional parameter which indicate that the permeability ${\mathbb {K}}$ has a dimension of length square which is normalized by length square. Hence particular parameter which is denoted by ${\mathbb {D}}a$ (nondimensionalized parameter) called Darcy number. It shows that if ${\mathbb {K}}$ goes to infinity (large value), the corresponding equation produces Stokes equation particularly this is the limiting case. The Darcy number represents the rate of percolation inside the porous medium which is proportional to permeability. So large Darcy number means large permeability and small Darcy number indicates small permeability. The Fig. 3a–f indicate the general solution comparison corresponding to the interface boundary condition which can bee seen from Eqs. (14, 15).

**Figure 4:**
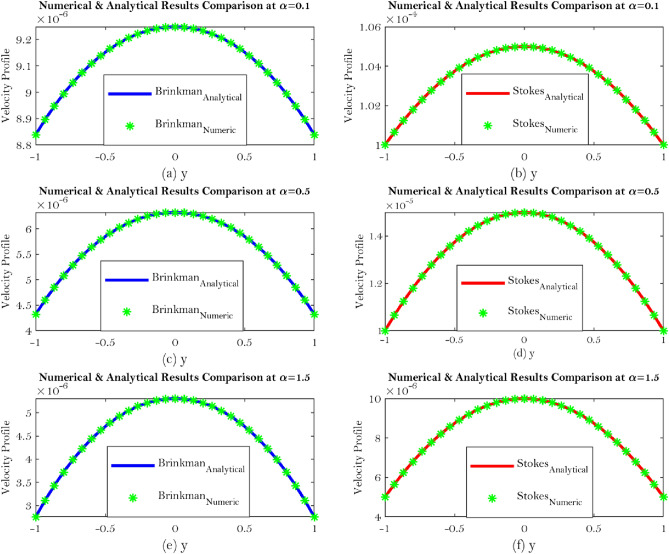
Shows velocity comparison at different value of the slip parameter $\alpha$ such as $\alpha = 0.1, \ 0.5 \ \hbox {and} \ 1.5$.

The use of Brinkman’s equation was preferred over the use of Beavers and Joseph’s boundary condition for flow in a channel over a Darcy porous layer by several writers owing to the compatibility of various orders between Brinkman’s equation and the Navier-Stokes equations. According to physical set-up to Brinkman’s equation has been addressed continuity in velocity and shear stress at the interface as well as recovering Beavers and Joseph’s findings by specifying the slip parameter $\alpha$. Such boundary condition emphasizes Brinkman’s equation which is a better acceptable model when the porous layer has a limited depth because it is more accurate. In the many evaluations of flow through and over porous layers there are several modifications of the Beavers and Joseph condition that have been presented. An extension to study the Beavers and Joseph condition to a problem of Synovial fluid flow through a porous layer. Equations (23$\longrightarrow$27) are calculated and depicted through Figs. 4a–f to show velocity comparison at different value of the slip parameter $\alpha$ such as $\alpha = 0.1, \ 0.5 \ \hbox {and} \ 1.5$.

**Figure 5:**
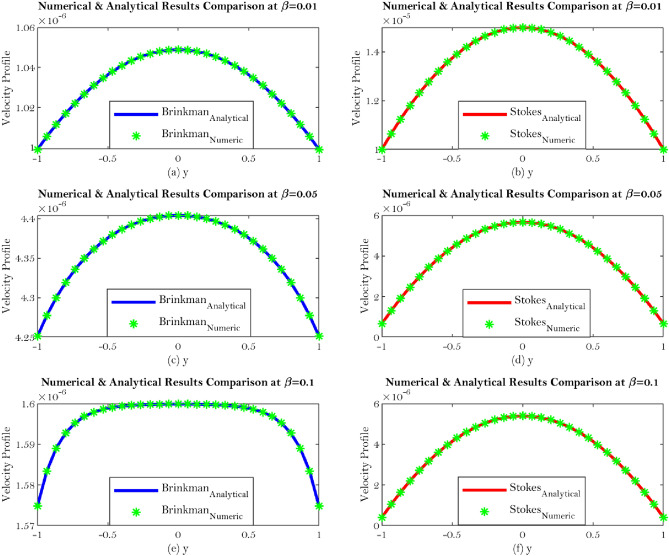
Shows velocity comparison at different value of the $\beta$ such as $\beta = 0.01, \ 0.05 \ \hbox {and} \ 0.1$.

The solution to Eqs. (14) and (15) under the given physical boundary conditions which lead to Eqs. (24) and (27) in case of non-dimensionalization of the problem with respect to velocity component. Such that the upper plate is analogous to Beavers and Joseph condition. The gap between upper and lower plates are from $-H$ to *H*, so size is 2*H*. We can fix at $y=-H$ with no slip or $y=H$ with Beavers and Joseph condition and then solve the complete domain. So in this case we used the symmetry condition with respect to $y=0$ which solve the problem in the proportion. Since $u^{*}$ is the function of *y* which shows that left hand side is completely function of *y* and $\partial p^{*}/\partial y=0$ and thus pressure is only a function of *x*. So each must be constant hence $\partial p^{*}/\partial x$ is constant which is analogous to clear flow scenario. Thus, the damping force due to the porous medium naturally when we non-dimensionalize, some parameter controlling the structure (like $\beta$). Figure 5a–f show velocity comparison at different value of the $\beta$ such as $\beta = 0.01, \ 0.05 \ \hbox {and} \ 0.1$.

**Figure 6:**
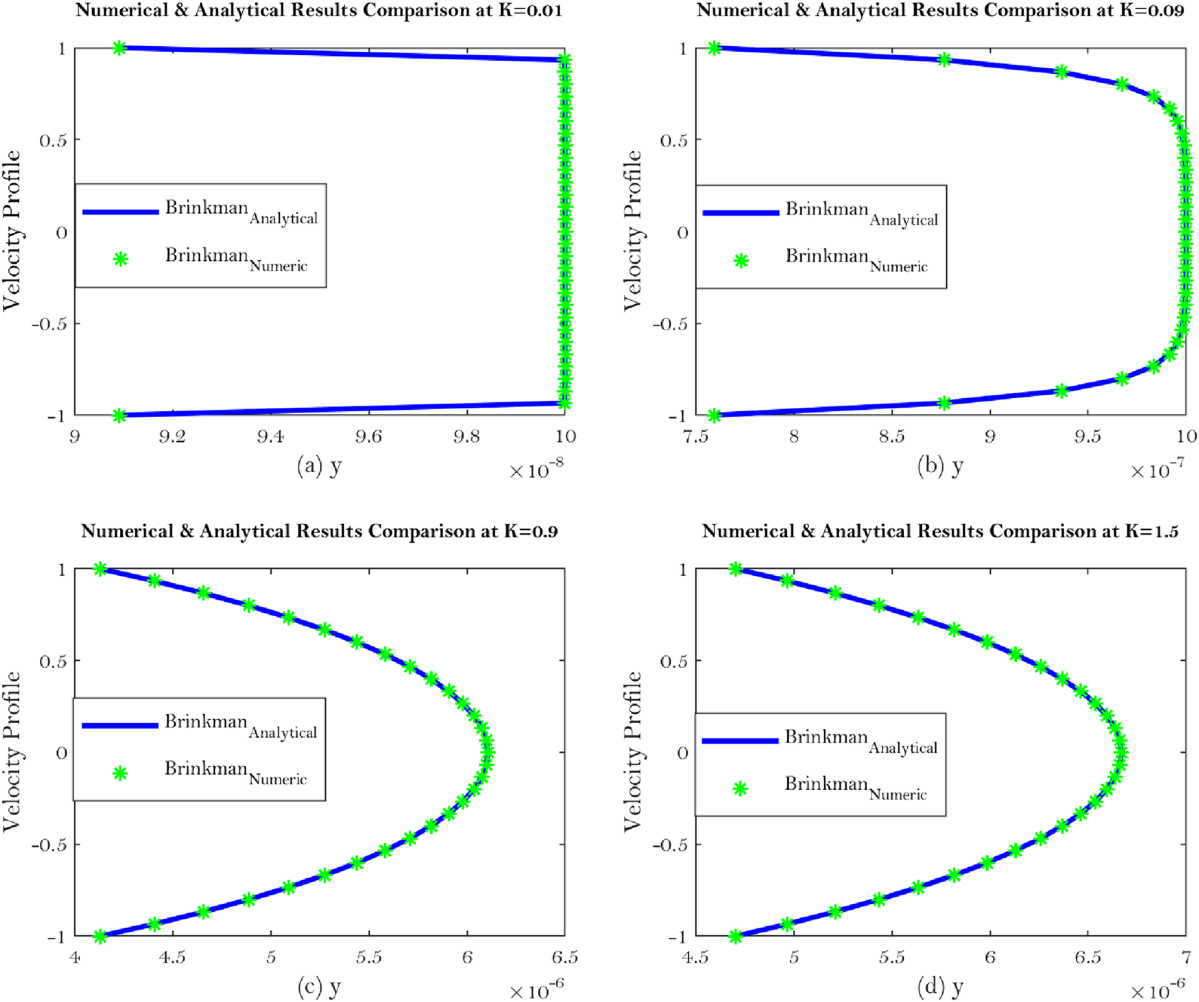
Shows velocity comparison at different value of the ${\mathbb {K}}$ for Brinkman’s equation.

**Figure 7:**
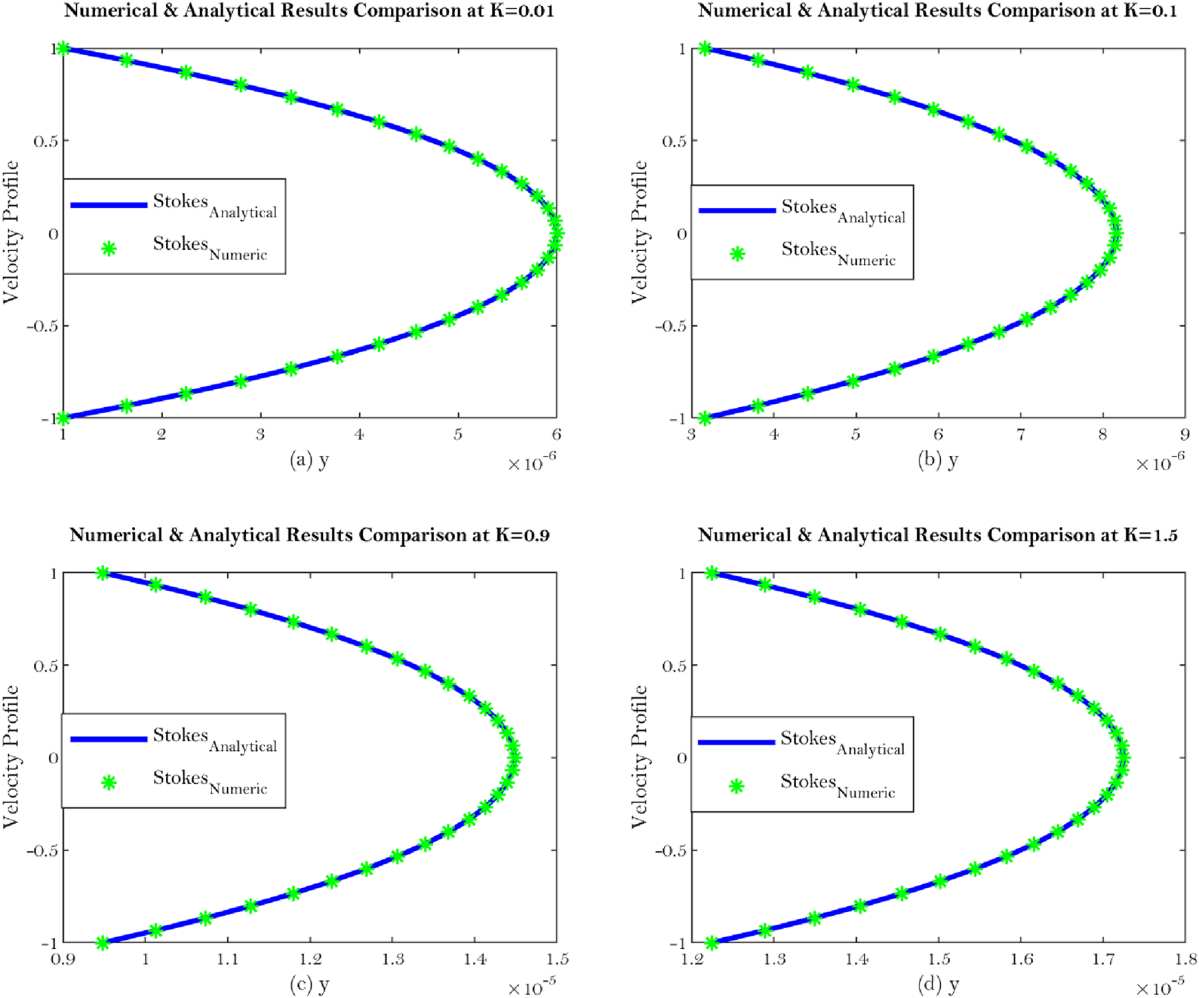
Shows velocity comparison at different value of the ${\mathbb {K}}$ for Stokes equation.

The Brinkman’s and Stokes equations for synovial fluid flow at well defined mathematically smooth boundary condition (Beavers and Joseph’s) at $y=H$ filled with porous material with uniform permeability ${\mathbb {K}}$ are used to assess the findings obtained by employing the analytical solution to these equations. By allowing the fluid permeability in the porous material area to vary (for different values of ${\mathbb {K}}$), the analytical solution may excellently fit to the numerical solution which can be seen from Figs. 6a–d, 7a–d.

**Figure 8:**
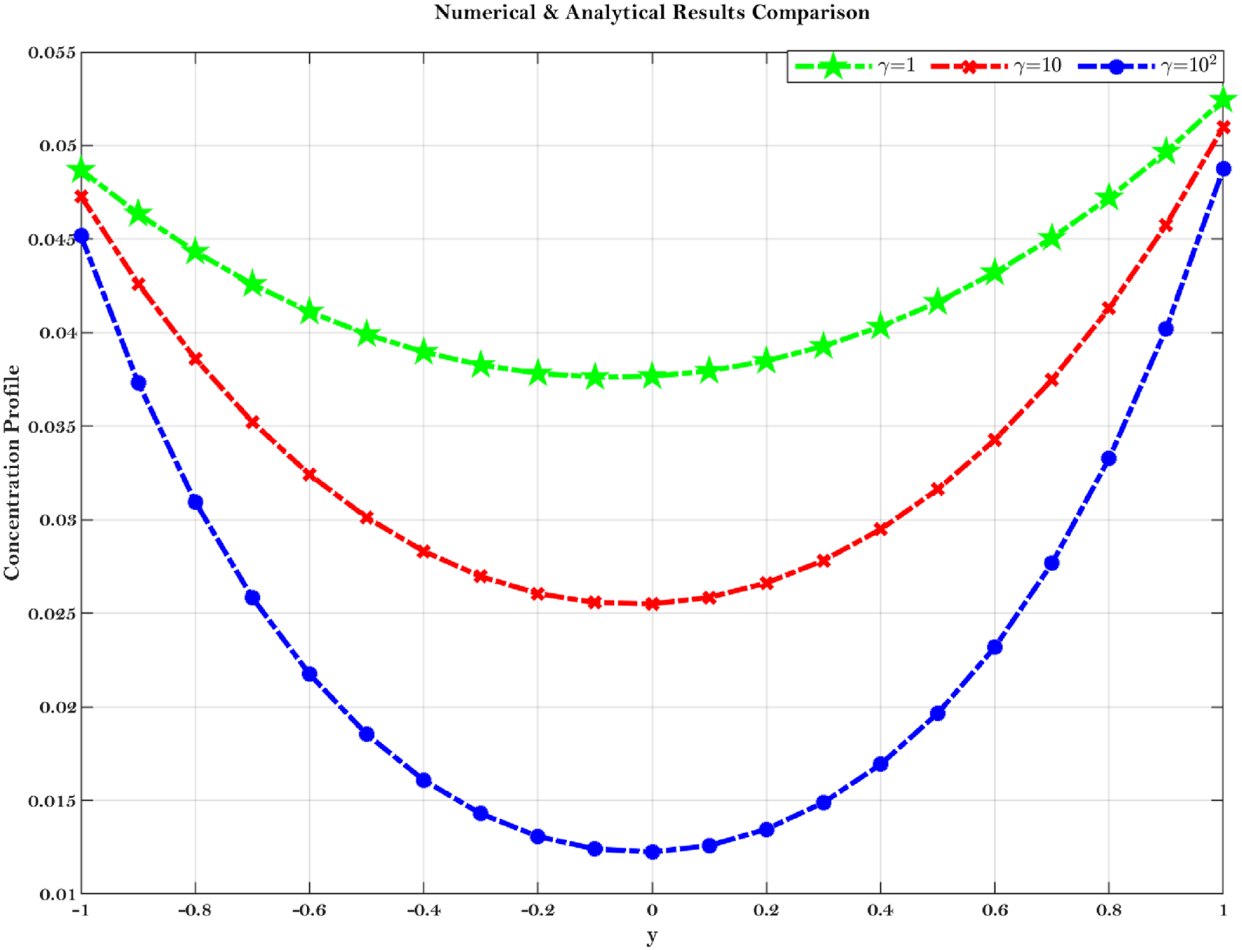
Shows concentration profile comparison at different value of the $\gamma$.

An analogous conclusion has been reached by^18,22–25^ in a laminar flow between two parallel plates by taking into account both the homogeneous and heterogeneous reactions of the solvent and solute in the solution. Diabetes patients have increased viscosity of plasma when compared to healthy ones and as a result the diffusion coefficient for diabetes patients is on average higher than that of healthy subjects^18,22–26^. The fluctuation of the mean concentration distribution with axial distance for various values of $\gamma$ is seen in the Fig. (8).

**Figure 9:**
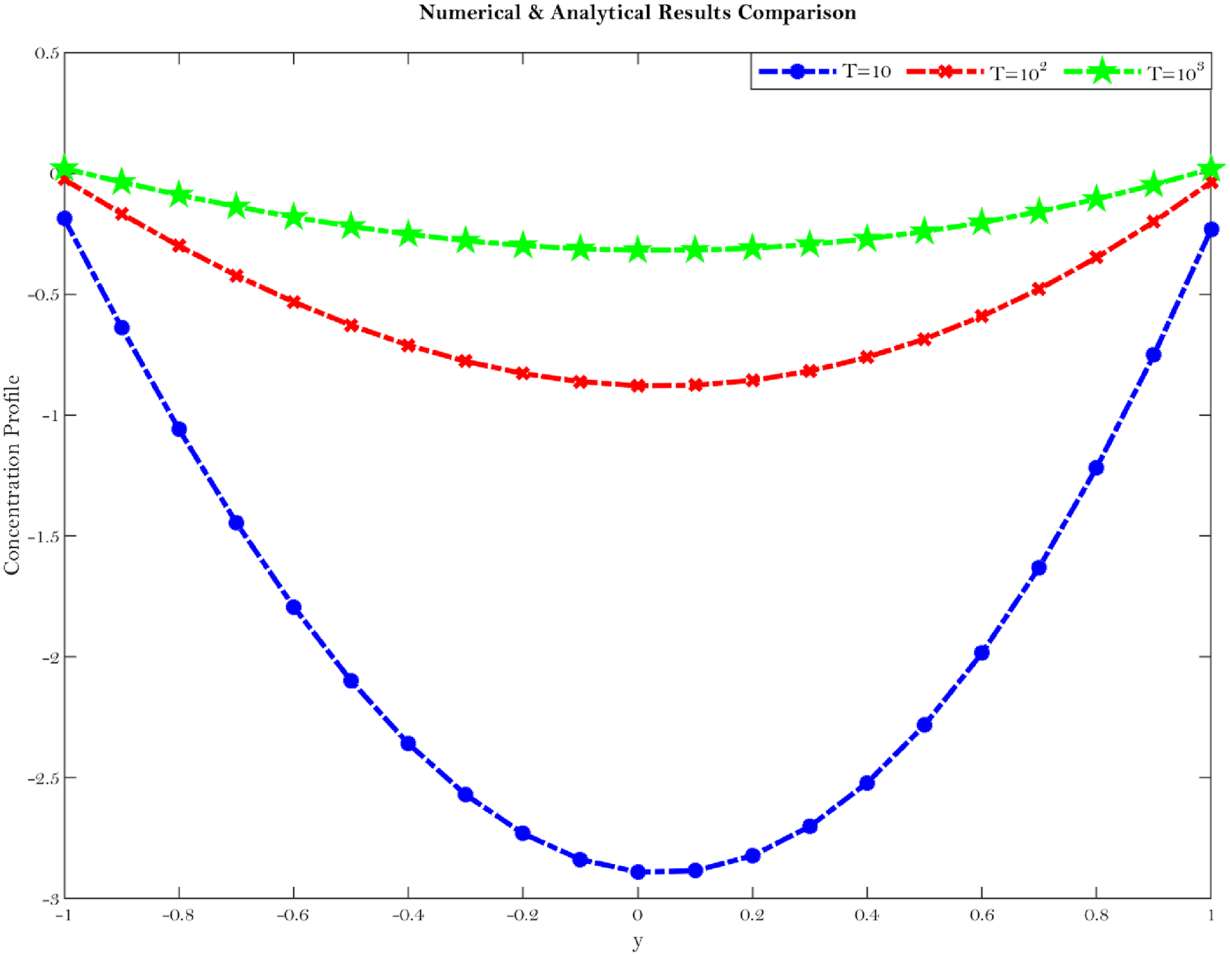
Shows concentration profile comparison at different value of the ${\mathbb {T}}$.

In this study, it has been shown that the mean concentration distribution decreases with increasing axial distance which can be seen from the Fig. (9). From Eqs. (16, 33) concentration study is done by using steady convection diffusion phenomena. This is because the key mechanisms involved in cartilage regeneration modelling cell migration, nutrient diffusion and depletion, extracellular matrix synthesis and degradation^18,22–26^. It is also discovered that the cells in the center region get greater nutritional support than the cells in the periphery area. It assists orthopedic surgeons in determining whether or not the joints are functioning properly by using the formula to validate their findings. As a result, the model for steady convective diffusion can be used to develop a mathematical model for articular cartilage regeneration in the future.

## Conclusion

The present investigation, the Stokes-Brinkman coupling is used to unite the fluid domain Stokes equation with the porous media Brinkman equation. Evidence suggests that this model accurately represents the knee joint^27–30^. The Beavers and Joseph slip conditions, which are a specific boundary condition for the synovial fluid, are used to solve the governing system of partial differential equations, and the results are presented here for the first time, illuminating the convective diffusion of the viscous flow along the articular surfaces between the joints. The mechanics of nutrient transport into the synovial joint are of interest, and hence an analytical solution is being developed, along with a numerical simulation (using the finite volume technique). The average concentration was found to be inversely related to both axial distance and time spent in the experiment^29,30^. Several graphs have been used to learn about the problem’s many facets, such as its velocity and concentration.

## Closing remarks

The current work addresses the use of a specific boundary condition for the flow of synovial fluid with concentration effects in synovial joints. The key findings of this research are as follows;

- Synovial fluid can be thought of a single component fluid, i.e homogeneous which indicates to use continuous approach. Physically, Brinkman and Stokes solutions suggest comparable velocity behaviour. The outcomes of this study will let us use an alternate equation to comprehend synovial fluid’s homogeneous velocity behaviour, which can be seen from Eqs. (26, 30) and Figs. 6a–d, 7a–d.
- Synovial fluid has the property of being in-compressible.
- The content of Hyaluronic acid (HA) in synovial fluid which occurs in the introduction to the model for synovial fluid has an effect on the mechanical reactions of the fluid.
- Large Darcy number means large permeability and small Darcy number indicates small permeability^29^, which can be seen from Fig. 3a–f.

## Data Availability

The data that support the findings of this study are available within the article.

## List of symbols

x, y: Coordinate system
*u*, *v*: Synovial fluid Velocity components along *x**y* directions
$\rho$: Synovial fluid density
$$\dfrac{D}{Dt}$$: Total derivative
$${\textbf{F}}$$: Body force
$$\mu $$: Viscosity
$$\mu ^{'}$$: Effective viscosity
${\textbf{K}}$: Permeability of porous medium
$$\alpha $$: Slip parameter
2*h*: Fluid film thickness
$${\textbf{C}}$$: Concentration
$${\textbf{P}}$$: Pressure
$${\bar{u}}$$: Average velocity
$${\textbf{D}}_{Diff}$$: Diffusion coefficient
$${\textbf{D}}a$$: Darcy number
$\gamma$: Non-dimensionalised parameter
*L*: Characteristic length
${\textbf{T}}$: Non-dimensionalised parameter
$A_{n}$: Area along north face
$A_{s}$: Area along south face
$\delta y$: Distance between nodes
$\delta y$: Distance between nodes
$\delta y_{PN}$: Distance between node *P* and face *N*
$\delta y_{SP}$: Distance between node *P* and face *S*
$a_{S}, a_{N}$: Constants along south and north faces
$a_{P}$: Centroid of control volume
